# Building early-larval sexing systems for genetic control of the Australian sheep blow fly *Lucilia cuprina* using two constitutive promoters

**DOI:** 10.1038/s41598-017-02763-4

**Published:** 2017-05-31

**Authors:** Ying Yan, Rebecca J. Linger, Maxwell J. Scott

**Affiliations:** 1 Department of Entomology and Plant Pathology, North Carolina State University, Campus Box 7613, Raleigh, NC 27695-7613 USA; 2 Fraunhofer IME-BR, Winchesterstr. 2, 35394 Giessen, Germany

## Abstract

Transgenic sexing strains (TSS) that carry conditional female lethal genes are advantageous for genetic control programs based on the sterile insect technique (SIT). It is desirable if females die early in development as larval diet is a major cost for mass production facilities. This can be achieved by using a gene promoter that is only active in embryos to drive expression of the tetracycline transactivator (tTA), the transcription factor commonly used in two-component TSS. While an embryo-specific promoter is ideal it may not be essential for assembling an effective TSS as tTA can be repressed by addition of tetracycline to the diet at larval and/or adult stages. Here we have investigated this idea by isolating and employing the promoters from the *Lucilia spitting image* and *actin 5C* genes to drive tTA expression in embryos and later stages. *L. cuprina* TSS with the tTA drivers and tTA-regulated *tetO-Lshid* effectors produced only females when raised on a limited tetracycline diet. The *Lshid* transgene contains a sex-specific intron and as a consequence only females produce LsHID protein. TSS females died at early larval stages, which makes the lines advantageous for an SIT program.

## Introduction

The ability to make transgenic insects has been invaluable for genetic analysis and also offers the potential for developing strains that have practical applications^1^. We previously developed efficient methods for germline transformation of the Australian sheep blow fly *Lucilia cuprina* ^2, 3^, a major pest of sheep^4^. This technology was used to create transgenic sexing strains (TSS) of *L. cuprina* for potential use in a genetic control program^5, 6^. Sexing strains provide a mechanism for sorting of sexes such that only males are released in the field. This is advantageous for sterile insect technique (SIT) programs as sterile females are ineffective as control agents and compete with females in the field for mating with sterile males^7, 8^. In an SIT program, insects are mass reared in factory, sterilized by ionizing radiation and released over a selected area in excess of the targeted population (usually at least 10-fold excess)^9, 10^. The Mediterranean fruit fly (*Ceratitis capitata*) SIT programs use a genetic sexing strain (GSS) that has a functional copy of a temperature sensitive lethal (*tsl*) gene translocated to the Y chromosome^7^. Females, which are homozygous for the *tsl* mutation, die when raised at the non-permissive temperature. Releases of sterilized males of *C. capitata* are 3–5 times more efficient at population suppression than bisexual releases under field conditions^11^. Disadvantages of the GSS are that it is semi-sterile and prone to breakdown during mass rearing as a consequence of recombination in males^7^.

The initial *L. cuprina* TSSs we developed carried a single transgene that consisted of a tetO_21_-hsp70 enhancer-promoter driving expression of tetracycline transactivator (tTA) transcripts that were sex-specifically spliced as they contained the regulated intron from the *Cochliomyia hominivorax transformer* (*Chtra*) gene^5^. Only the female transcript encoded tTA protein, which is a transcription activator that binds to the tetO sequence^12^. Binding of tTA to tetO is inhibited by tetracycline^12^. When the TSSs were raised on standard diet without tetracycline, autoregulation of tTA gene expression led to very high levels of tTA and female-specific lethality^5^. The lethality is thought to be due to “transcriptional squelching” and occurs at the late larval/pupal stages. The TSSs were based on a similar autoregulated sex-specific genetic system developed earlier for tephritid fruit flies^13, 14^.

Although the TSSs produced only males, which is advantageous for SIT programs as discussed above, there would be little savings in larval diet costs due to the late stage of female lethality. Consequently, we recently developed sexing strains of *L. cuprina* that carried a two-component transgenic embryonic sexing system (TESS)^6^ that was similar to systems developed earlier for tephritid fruit flies^15, 16^. One of the transgenes consisted of tTA driven by the promoter of the *L. sericata bottleneck* cellularization gene that is expressed in the early embryo^17^. The “effector” transgene in the TESS was the *L. sericata hid* cell death gene driven by tetO_21_-hsp70 enhancer-promoter^6^. Further, the *Lshid* gene was interrupted by the *Chtra* sex-specific intron and, as a consequence, only the female transcript encoded functional HID protein. In the absence of tetracycline, tTA activated expression of the *Lshid* proapoptotic gene, leading to death of the female embryo.

Ideally, the promoter driving tTA in a TESS should only be active in the early embryo and not at later stages of development. If so, it should be possible to rear larvae and adults on diet without tetracycline. This would be important as females fed tetracycline would be expected to pass on the antibiotic to their offspring, which would inhibit tTA binding to DNA in the developing embryo. However, when the *L. cuprina* TESS were raised on diet that lacked tetracycline, half of the females died before the first egg collection and the remainder produced few eggs^6^. We found that if the adult diet was supplemented with a low concentration of tetracycline for the first 2 days after eclosion, that the females survived, produced eggs and their female offspring died at the embryo stage. The latter indicated that with this limited tetracycline-feeding regimen, little tetracycline was passed on to the embryo from the mother. These results suggested that it might not be necessary to find an embryo-specific promoter to build a TSS with early stage female lethality, if a suitable tetracycline-feeding schedule can be established.

The goal of this study was to determine if TSSs could be made using promoters from genes active in the embryo and also later developmental stages for controlling expression of tTA. In *Drosophila melanogaster*, the *actin5C* (*Dmact5C*) gene is strongly expressed throughout development, whereas the *spitting image* (*Dmspt*) gene is expressed at low levels at most stages but higher in embryos and adult females^18^. *Drosophila spt* is a paralog of the zygotic cellularization gene *serendipity alpha* (*sry-*α)^19^. The SPT and SRY-α proteins are members of the Vinculin/a-Cateninin superfamily and have redundant actin-regulating activity. *spt* RNA is maternally provided whereas *sry-*α is expressed in the zygote at around the time of cellularization^19^. Orthologs of the *spt* and *sry-*α genes were found in all *Drosophila* species genomes but only *spt* was found in other insects^19^. Here we determine if TSS can be made using promoters from the orthologous calliphorid genes, *Lcact5C* and *Lsspt*.

## Results

### *tTA* expression at different developmental stages in driver lines

The *Lsspt* and *Lcact5C* gene promoters were isolated using a similar strategy as used previously for the *L. sericata bnk* and *C. hominivorax slam* cellularization gene promoters^17^. Firstly, orthologs were identified in *L. sericata* and *L. cuprina* transcriptomes^20, 21^. In pairwise comparisons, the LsSPT protein was 41% identical to the *D. melanogaster* SPT protein but 31% identical to SRY-α. Further, when multiple sequence alignment and neighbor joining tree analyses were performed with dipteran SPT and Drosophila SRY-α proteins, LsSPT clustered with SPT proteins (Figs S1 and S2). An ortholog of *D. melanogaster* SRY-α was not found in the *L. sericata* or *L. cuprina* transcriptomes or *L. cuprina* genome^22^. The *L. cuprina* ACT5C protein was 100% identical with *D. melanogaster* ACT5C but also showed high identity with other *D. melanogaster* actins such as ACT42A (99%) and ACT87E (97%). In the *D. melanogaster* and *L. cuprina* genomes, the *act5C* gene is very closely linked to the CG4020 and CG12236 genes. Thus on the basis of amino acid conservation and genetic synteny, the gene we identified as *Lcact5C* is likely to be the ortholog of *Dmact5C*. To identify the probable start sites of transcription of the genes, we next performed 5′ RACE experiments with RNA from 2–3 h embryo, which is around the time of cellularization.

Since the project began before the release of the *L. cuprina* genome sequence^22^, the GenomeWalker Kit (Takara) was used to isolate upstream flanking sequences for the *Lsspt* and *Lcact5C*genes. In the *Lsspt* GenomeWalk, a 2.4 kb product was obtained, which was cloned and the DNA sequence was determined. The sequence overlapped with the *Lsspt* transcript sequence to yield 2.15 kb of upstream flanking sequence and a first exon that contains a 267 bp untranslated region upstream of the translation start codon. In the *Lcact5C* GenomeWalk, several overlapping products were obtained, which were cloned and the nucleotide sequences determined. The assembled overlapping sequence contained 1275 bp of upstream flanking, a 110 bp noncoding first exon, a 2662 bp first intron and 45 bp of the second exon. The assembled sequences were searched for TAGteam motifs, which are commonly found in the promoters of genes expressed early in development in *D. melanogaster* ^23^. The *Lsspt* upstream flanking sequence had one TAGteam motif 251 bp upstream from the 5′ end and single TAGteam motif occurs within the first intron of the *Lcact5C* gene. The *Lsspt* and *Lcact5C* first exons begin with the sequences TCAGGT and TCATTA, which partially match the consensus sequence for initiator motif (Inr) of *Drosophila* core promoters (TCAG/TTC/T)^24^. The *Lcact5C* promoter contains a TATA-box sequence (TTATAA) 30 bp upstream of the likely transcription start site (Fig. S3). The *Lsspt* promoter does not have a TATA-box sequence close to the likely start of transcription.

**Figure 1:**
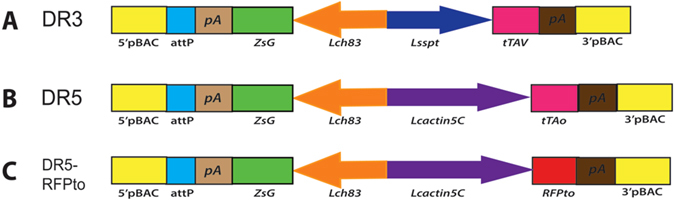
Schematic illustration of the driver constructs. All constructs contain the ZsGreen marker driven by the *Lchsp83* gene promoter, a *phiC31* attP site and 5′ and 3′ *piggyBac* ends. Expression of *tTA* is controlled by the *Lsspt* promoter in DR3 (**A**) and the *Lcact5C* promoter in DR5 (**B**). The *Lcact5C* promoter was also used to drive expression of the dtTomato (RFPto) reporter gene (**C**).

**Figure 2:**
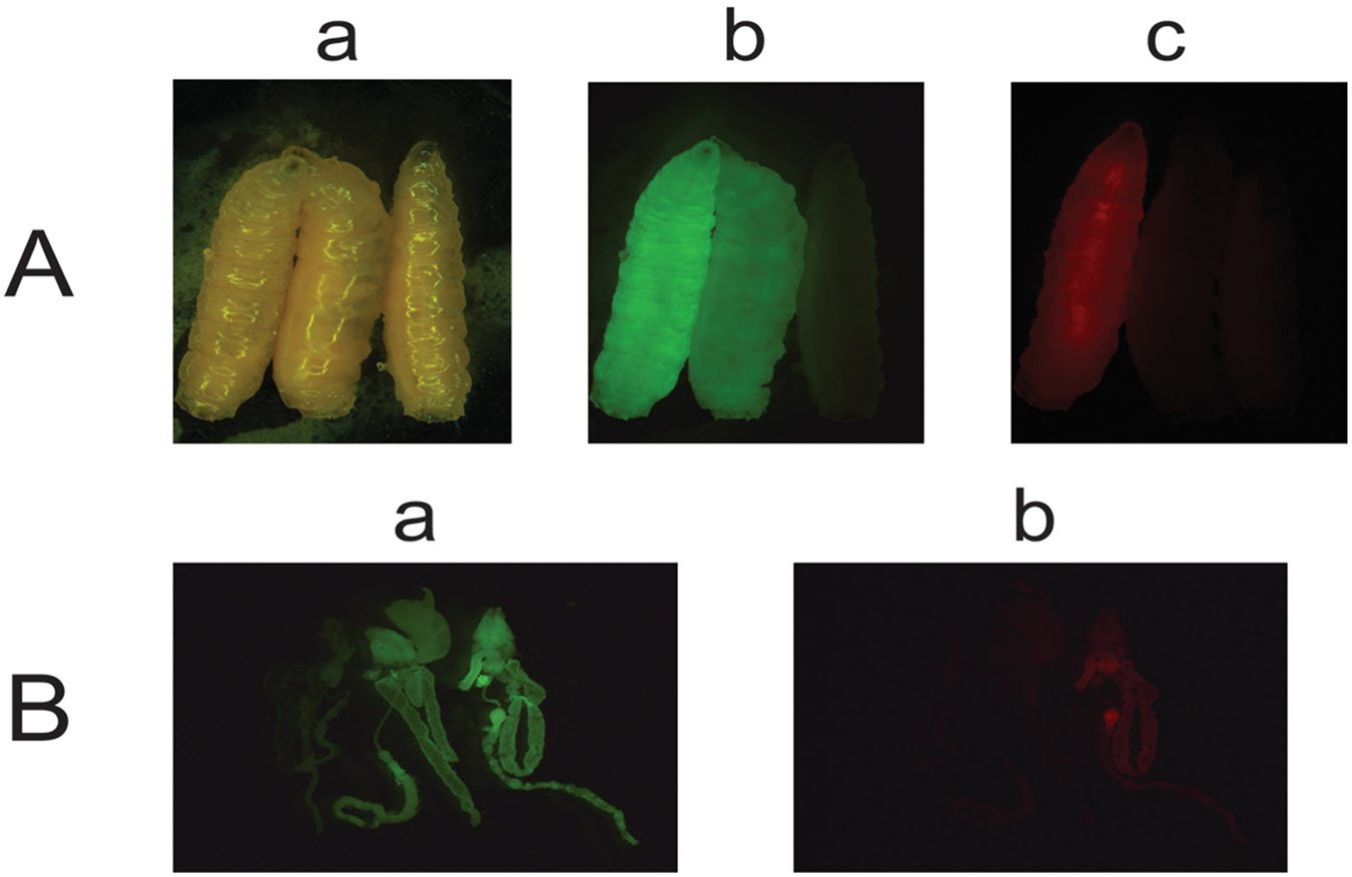
The *Lcact5C* gene promoter directs expression of the tdTomato (RFPto) marker in most cells but predominantly in gut. Whole body (**A**) and dissected gut tissues (**B**) from *L. cuprina* 3^rd^ instar marked with constitutively expressed fluorescent protein marker genes. The larvae in (**A**) were from *pBac-Lch83 promoter-ZsGreen -Lcactin5C promoter-RFPto* transformed homozygous line (left), *pBac-Lch83 promoter-ZsGreen* transformed homozygous line (middle) and wild type (right), respectively, and the photos were taken under white light (a), GFP filter (b) and DsRed filter (c). The dissected gut tissues in (**B**) were from wild type (left), *pBac-Lch83 promoter-ZsGreen* transformed homozygous line (middle) and *pBac-Lch83 promoter-ZsGreen -Lcactin5C promoter-RFPto* transformed homozygous line (right), and the images were taken under GFP filter (a) and DsRed filter (b).

To assay for *Lcact5C* promoter activity, the upstream flanking sequence, first exon, first intron and part of the second exon through the start of translation was fused in-frame to the tdTomato (RFPto) fluorescent protein reporter^25^ and cloned into a *piggyBac* transformation vector with a constitutively expressed *Lchsp83-ZsGreen* marker gene^3^ (Fig. 1). Three transgenic lines were obtained by *piggyBac*-mediated germ-line transformation and bred to homozygosity. The transgenic larvae showed a low level of red fluorescence throughout the body (Fig. 2). Stronger fluorescence was detected in the gut. This pattern of expression was similar in all lines and thus likely reflects the activity of the *Lcact5C* promoter.

**Figure 3:**
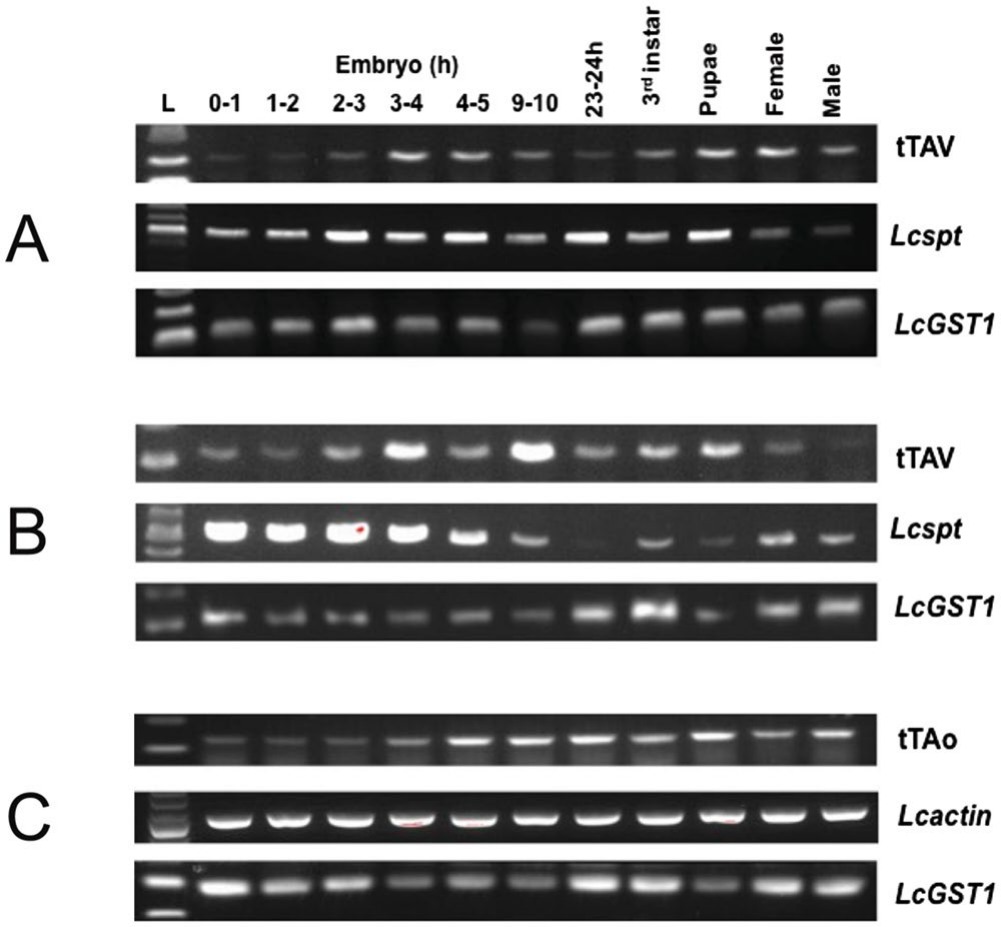
tTA expression at different developmental stages in transgenic driver lines. RNA was isolated from the lines DR3#2, DR3#4 and DR5#4 and analyzed by RT-PCR. The sizes of RT-PCR products are: 206bp for *tTAv*, 165bp for *tTAo*, 515bp for *Lcspt*, 495bp for *Lcact5C* and 121 bp for *LcGST1*.

The DR3 and DR5 driver constructs contain the *Lsspt and Lcact5C* gene promoters respectively, upstream of the *tTAv* (DR3) or *tTAo* (DR5) coding regions (Fig. 1). *tTAo* is a version of tTA that is codon-optimized for translation in *L. cuprina* ^6^. DR3 and DR5 also contain the *Lchsp83*-*ZsGreen* marker gene and *piggyBac* left and right ends. Fourteen DR3 lines were obtained from 119G_0_ adults (63 males, 56 females) by *piggyBac*-mediated germline transformation. Nine autosomal lines were retained that carried a single transgene and were homozygous viable and fertile. Since the goal was to use the DR3 lines to make early-acting sexing strains, RNA was isolated from a single collection of 2–3 h embryos and *tTAv* transcript measured by qRT-PCR analysis (Fig. S4). Based on these results, two lines, DR3#2 and DR3#4 were selected for further analysis and for crossing with *tetO-Lshid* effector lines. Three DR5 *L. cuprina* transgenic lines were obtained from 64G_0_ adults (34 males and 30 females). One line was weak and could not be maintained. The remaining lines, DR5#2 and DR5#4, were found to be X-linked and only the DR5#4 line was homozygous viable. Further, as DR5#2 adult males have very low viability and fertility, the line was maintained by crossing heterozygous females with males from the wild type parental strain. The selected DR3 lines (#2 and #4) and DR5 lines (#2 and #4) were analyzed for *tTA* expression at different developmental stages. RNA was isolated from pre-cellular embryos (0–1 h), embryos around the time of cellularization (1–2 and 2–3 h) and after cellularization (3–4, 4–5 and 9–10 h), third instar, pupae and young adults. With the DR5#2 line we were unable to distinguish heterozygous from wild type embryos due to maternal inheritance of the ZsGreen marker and consequently *tTAo* expression was not examined in embryo stages (Fig. S5). *Lcspt* and *Lcact5C* amplification products were detected at all stages, although expression of *Lcspt* appeared to be higher in embryos than later stages. Amplification products were readily detected in pre-cellular embryos (0–1 h), indicating maternal expression (Fig. 3). In the DR3 lines, the pattern of expression of *tTAv* was similar to *Lcspt*, with amplification products detected at all stages. *tTAo* was expressed at all stages examined in the DR5 lines (Figs 3 and S5).

### Female-specific lethality of double-heterozygous progeny for transgene combinations

**Figure 4:**
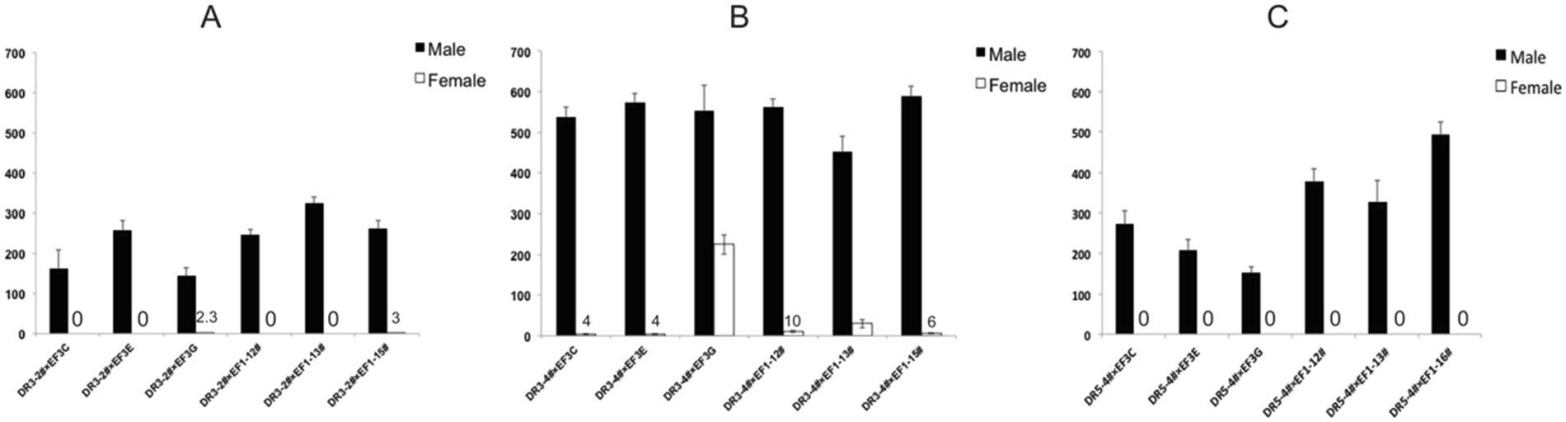
Female-specific lethality of double heterozygous lines. Homozygous DR3#2 (**A**) and DR3#4 (**B**) males were crossed with virgin females from each of the indicated homozygous effector lines. For the X-linked DR5#4 line (**C**), virgin females were crossed with males from each homozygous effector line. The offspring of the crosses were raised on diet without tetracycline. Error bars show the standard error of the mean (n = 3).

In an initial screen to assess the effectiveness of the driver lines, selected DR3 and DR5 lines were crossed with homozygous “effector” EF1 and EF3 lines and the number of adult male and female offspring counted (Fig. 4). The EF1 and EF3 transgenes express the *Lshid* cell death gene under control of the *tetO*_*21*_ *-Lchsp70* enhancer-promoter^6^. EF3 contains the wild type version of *Lshid* whereas EF1 has the more active version that has the two conserved MAPK phosphorylation sites changed to encode Alanine. In both effector constructs, the sex-specific first intron from the *Chtra* gene was inserted immediately downstream of the ATG translation start codon. Consequently, only female *Lshid* transcripts code for functional protein. Thus activation of transcription by tTA would lead to death of female offspring from the DR/EF crosses. All of the driver lines were effective at reducing female viability (Fig. 4). No female offspring were obtained from crosses with DR3#2 and four of the effector lines (Fig. 4A). The DR3#4 line was slightly less effective, as some female offspring were obtained from all crosses (Fig. 4B). Nevertheless, female viability was reduced by over 98% in three of the crosses. No female offspring were obtained from crosses with the DR5#4 line (Fig. 4C).

### Female-specific and staged lethality of strains homozygous for both driver and effector transgenes

**Figure 5:**
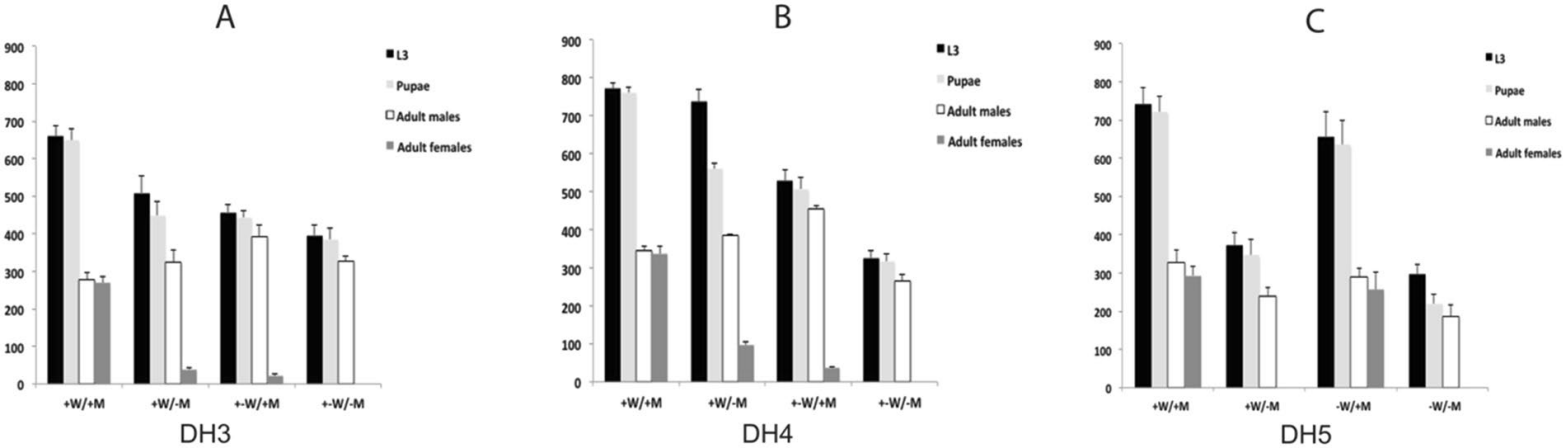
Female-specific lethality of double homozygous strains. DH3 (**A**), DH4 (**B**) and DH5 (**C**) were raised under different tetracycline feeding conditions. +W: adults given water with 100 μg/mL tetracycline from day 1 (D1) to D8 after pupal eclosion; +−W: water with limited tetracycline (3 μg/mL D1 to D2, then switched to water without tetracycline from D3 to D8); −W: water without tetracycline from D1 to D8; +M: larval diet (93% ground meat) with 100 μg/mL tetracycline;-M: meat without tetracycline. Eggs collected from 8 pairs of adults from each DH strain and error bars show the standard error of the mean (n = 3).

Strains homozygous for both transgenes, which we call double-homozygous (DH) strains, were obtained by selecting for larvae that strongly express red and green markers. The DH strains were raised on diet supplemented with tetracycline (100 μg/mL). DH3 combines DR3#2 with EF3E, DH4 combines DR3#4 with EF1–12 and lastly DH5 combines DR5#4 with EF1–12. On maintenance diet with tetracycline in adult water (+W) and larval diet (+M), all DH strains produced equal numbers of adult males and females (Fig. 5). To induce female lethality early in development, it is desirable if mothers do not pass on tetracycline to their offspring^6, 26^. However, the DH3 and DH4 strains produced very few eggs if the adults were not fed tetracycline. It was then necessary to determine a limiting tetracycline feeding regimen for adults that was sufficient to maintain female viability and egg production. We had previously found that DH strains that contain the *Lsbnk-tTA* driver required a low concentration of tetracycline (1–3 μg/mL) for the first 2 days after eclosion^6^. Similarly, we found that a dose of 3 μg/mL tetracycline for both DH3 and DH4 strains for the first 2 days after eclosion was sufficient. Under these limiting tetracycline conditions (+−W), DH3 and DH4 strains produced no female offspring if larvae were raised on diet without tetracycline (-M) (Fig. 5A,B). Further, females appeared to die before the third instar stage (L3) as the number of adult males obtained was similar to the total number of pupae and L3. Consistent with this interpretation, very few females were obtained if the larval diet contained tetracycline (+−W/+M), suggesting most of the females were dying at the embryo or very early larval stages (Fig 5A,B). If females were only dying at the embryo stage, feeding adults a high dose of tetracycline (100 μg/mL) (+W/−M) would be predicted to be sufficient to rescue viability. However, most females died, suggesting death can also occur at later stages.

Although DR5#4 adult females express *tTAo* (Fig. 3C), DH5 adults could be maintained on diet without tetracycline and females readily laid eggs. No female offspring were obtained if the larval diet also lacked tetracycline (−W/−M) (Fig. 5C). However, females were fully viable if the larval diet contained tetracycline (−W/+M), suggesting death is largely at the larval stages. Further, female offspring were not rescued if the parental generation was fed a high level of tetracycline but the larval offspring were raised on diet without tetracycline (+W/−M).

**Figure 6:**
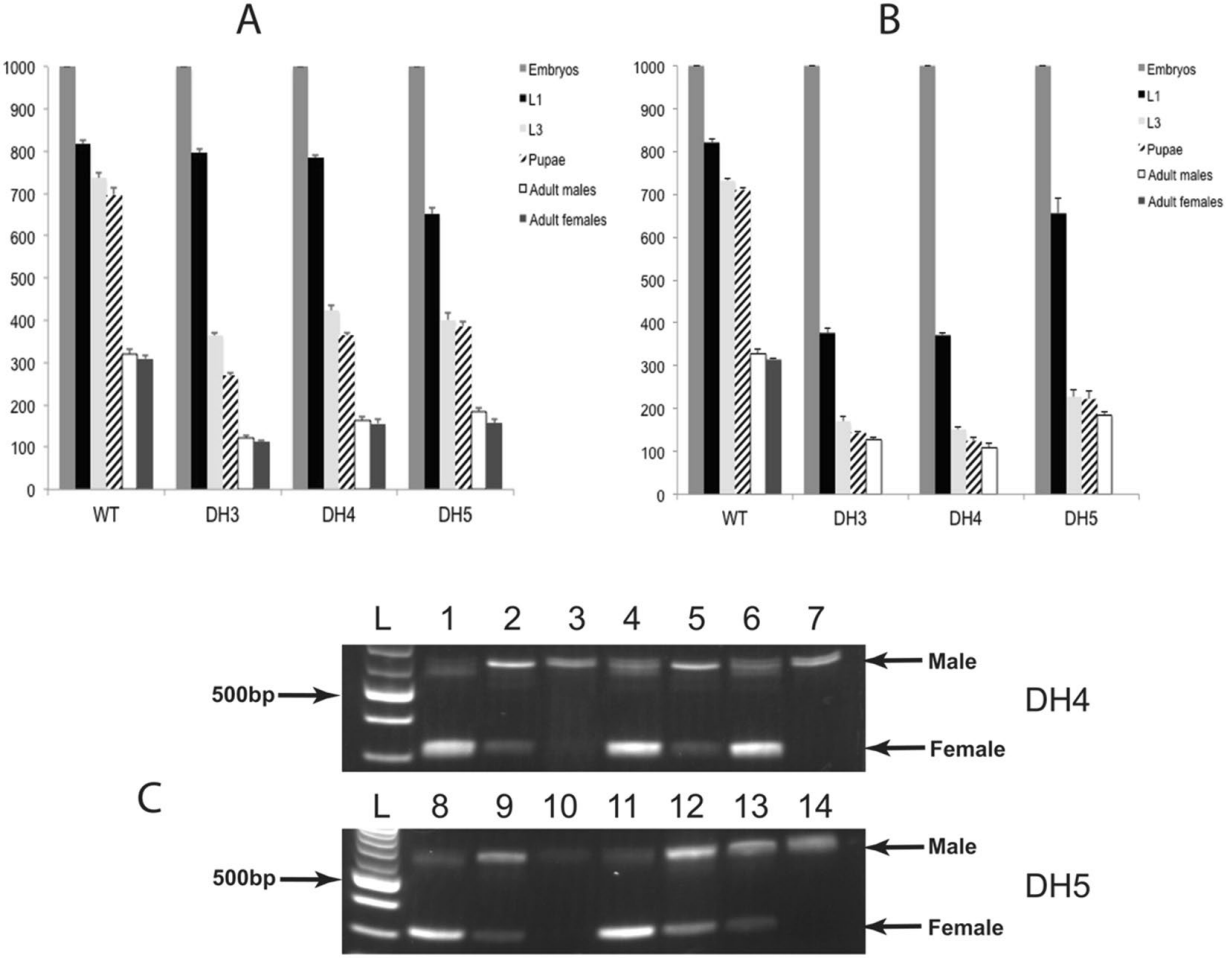
Staged lethality of double-homozygous transgenic sexing strains. DH3, DH4, DH5 and WT flies reared on diet with a high concentration of tetracycline (**A**) and without (WT, DH5) or limited tetracycline (+−W, DH3, DH4) (**B**). 1000 embryos were collected and the number of L1, L3, pupae, adult males and adult females were recorded. Error bars show the standard error of the mean (n = 3). The relative proportion of male and female larvae at different stages was determined by RT-PCR (**C**). With the *Lctra* primer pair used, 636 bp and 325 bp products were obtained from male and female transcripts respectively. RNA was isolated from WT, DH4 and DH5 strains. cDNA prepared from RNA from adult WT male and female RNA was mixed at ratios of 1:1 (lanes 1,8), 10:1 (lanes 2,9) and 100:1 (lanes 3,10) prior to amplification. Samples from DH4 were: 4) 100 L1 under ++W condition; 5) 100 L1 under +−W condition; 6) 100 L2 under ++W/+M condition; 7) 100 L2 under +−W/−M condition. Samples from DH5 were: 11) 100 L3 under ++W/+M condition; 12) 100 L1 under −W condition; 13) 100 L2 under −W/−M condition; 14) 100 L3 under −W/−M condition. L, DNA ladder.

To confirm the stage of lethality, 1000 eggs were collected from the DH strains and the number of hatched first instar, third instar, pupae and adult males and females were counted. For reference, the strains were fed a maintenance level of tetracycline (100 μg/mL) (Fig. 6A). For the wild type control (WT), the DH3 and DH4 strains, approximately 80% of the embryos hatched into larvae. The hatch rate was lower in the DH5 strain (65%) (Fig. 6A). In WT, 85% of the first instar developed into pupae. In the DH strains, the proportion of larvae that developed into pupae (34%, 46% and 59% for DH3, DH4 and DH5 respectively), was significantly less than wild type (P < 0.01). Equal numbers of adult males and females were obtained for WT and DH strains reared in diet containing tetracycline, while insects raised in diet without tetracycline (DH5), or limited tetracycline (+−W) (DH3, DH4), produced no adult females (Fig. 6B). Significantly, for DH3 and DH4, nearly half as many first instar larvae were obtained on the limited tetracycline diet compared to the high tetracycline diet. This suggests that most of the DH3 and DH4 females died at the embryo stage. For DH5, the embryo hatch rate was similar on diet with or without tetracycline (Fig. 6B). However, the number of third instar and pupae obtained on diet without tetracycline was about half that obtained on diet with tetracycline. This suggested that most of the females were dying between the hatched first instar and wandering third instar stages. Under a limited tetracycline feeding regimen, DH5 had higher male production (185.6 ± 8.2 males/1000 embryos) than DH3 (127.6 ± 4.8) or DH4 (108.3 ± 10.2) but less than wild type (327.6 ± 11.1).

We next performed RT-PCR analysis of RNA isolated from different stages to confirm when females died (Fig. 6C), as performed previously^6^. In brief, *Lctra* primers amplify a larger product from male template than female cDNA. For comparison, cDNA made from adult male and female RNA was mixed at a 1:1, 10:1 and 100:1 male:female ratio. At a 1:1 ratio the smaller female product predominates (Fig. 6C, lanes 1,8). At a 10:1 ratio, the main product is from males but the smaller female product was detected (Fig. 6C, lanes 3,10). When raised on diet with high levels of tetracycline, the predominant product detected in DH4 and DH5 larvae is the smaller female fragment (Fig. 6C, lanes 4,6,11). If DH4 adults were fed a limited tetracycline diet and the larval offspring raised without tetracycline (+−W, -M), the male product is the predominant product in first instar (lane 5) and the female band was not detected in L2 (lane 7), thus confirming that most females had died at the embryo stage. When DH5 was raised on diet without tetracycline, the female band was detected in L1 (lane 12) and L2 (lane 13) but not L3 stages (lane 14). The intensity of the female band relative to the male band was less in L1 than in larvae raised with tetracycline and in L2 the male band was predominant. These results indicate females are dying at all larval stages but most females have died before the L3 stage.

**Figure 7:**
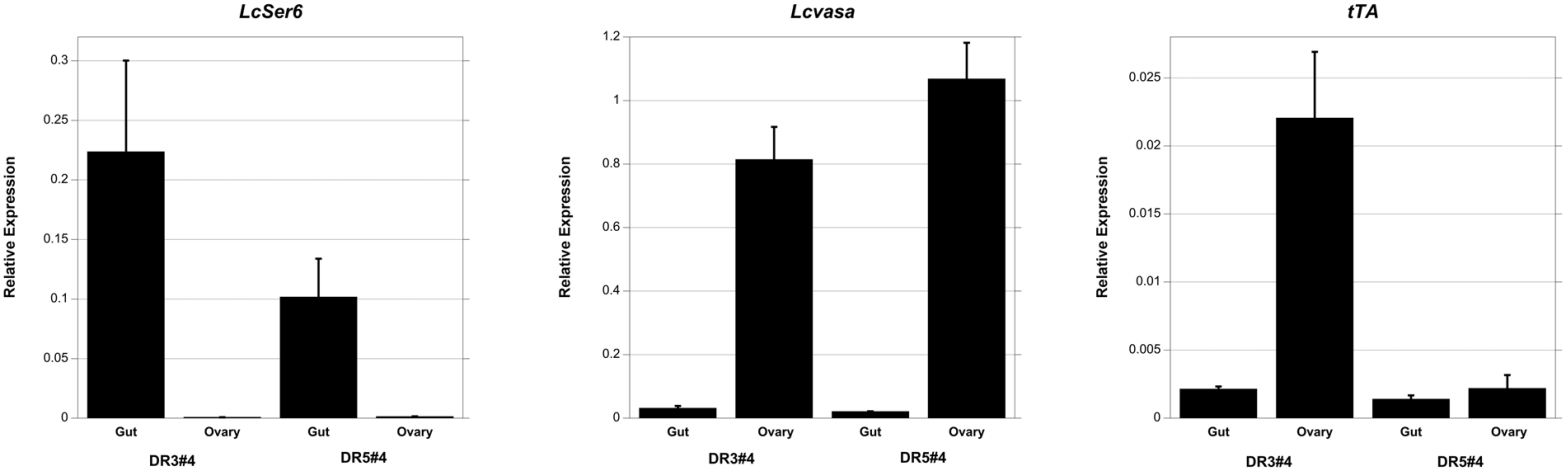
*tTA* expression in gut and ovary tissue from adult females. Relative expression of *LcSer6* (left panel), *Lcvasa* (center panel), *tTAv* (DR3) and *tTAo* (DR5) (right panel) transcripts in total RNA isolated from adult female gut and ovary tissue dissected from DR3#4 and DR5#4 lines. Transcript levels were normalized to *Lcalpha-tubulin*. Mean relative expression +/− standard error from three biologically independent replicate qRT-PCR experiments are shown.

Lastly, we next investigated why DH5 adults could be maintained on diet without tetracycline whereas it was necessary to feed DH3 and DH4 adults a low dose of tetracycline for the first two days after eclosion. One possible explanation is that *tTAv* is expressed at relatively high levels in immature ovaries in the DH3 and DH4 females, activating *Lshid* expression and thus inducing apoptosis. To address this prediction, gut and ovary tissue was dissected from one-day old adult females from the DR3#4 and DR5#4 lines. Quantitative RT-PCR analysis was then performed on total RNA extracted from the tissues to measure the relative levels of *tTAv* and *tTAo* transcripts. To control for dissection accuracy, we also measured the transcript levels from the *L. cuprina* othologs of the *D. melanogaster Ser6* and *vasa* genes^22^, which are mostly expressed in digestive tissue and ovaries respectively in *D. melanogaster* adult females^18^. The ovary is small and not fully developed in young *L. cuprina* females that have not been fed protein^27^. We found that *LcSer6* was expressed more highly in gut than ovary, whereas *Lcvasa* was expressed at higher levels in ovary than gut (Fig. 7). In DR3#4 females, *tTAv* was expressed at significantly higher levels in immature ovary than gut (Fig. 7, upper panels) (P < 0.05). In DR5#4 females, *tTAo* was expressed at similar levels in gut and immature ovary (Fig. 7, lower panels). In support of our hypothesis, *tTAo* was expressed at an order of magnitude lower level in DR5#4 ovaries than *tTAv* RNA in DR3#4 ovaries.

## Discussion

In this study we have developed *L. cuprina* early-larval TSS using gene promoters that are active throughout development to control expression of tTA. The TSS could be used in a genetic control program for *L. cuprina*, which is a significant economic pest in Australia and New Zealand^4^. The eradication of the New World screwworm (*C. hominivorax*) in North and Central America using SIT led to a major effort in the 1970s–1980s to develop field female killing (FFK) strains for genetic control of *L. cuprina* ^4, 10^. Although a field trial produced promising results, the program was abandoned due to cost and strain instability. The latter was a consequence of recombination in males leading to breakdown of Y:autsosome translocations in the FFK strains^28^. The TSS developed here could be used for an SIT or fertile male (RIDL) genetic control program. For the latter, it is advantageous if females die the embryo or early larval stages, as it is the late stage larvae that cause most of the economic damage. Further, for either SIT or RIDL, early stage female lethality would result in significant savings in diet costs as the majority of the larval diet is consumed by the third instar, which is the final instar before pupation. The TSS should be more stable than the FFK strains but strain stability remains to be evaluated under mass rearing conditions.

The *Lsspt* promoter was active throughout development including pre-cellular embryos, indicating maternal expression. Similarly, in *D. melanogaster spt* RNA is maternally provided^19^. DH3 and DH4 (carry a *Lsspt-tTA* transgene) females died mostly at the embryo stage under non-permissive conditions. However, to obtain sufficient egg laying it was necessary to feed adults a diet with a low concentration of tetracycline for the first two days after eclosion. This is probably a consequence of *tTA* expression in pupae and young adults leading to induction of *Lshid*. Indeed, quantitative RT-PCR analysis showed that *tTAv* is expressed in immature ovaries in young adult females at significantly higher levels than in gut. Similar to our results, Schetelig and colleagues found that females from a Mexican fruit fly TSS had nonvitellogenic oocytes and produced no offspring unless tetracycline was added to the diet for the first few days after eclosion^29^. Sterility was thought to be due to tTA expression in adult females activating expression of the *tetO-hid* effector gene. Although the *Lcact5C* promoter was active in pupae and adults, it was not necessary to feed DH5 (carry a *Lcact5C-tTAo* transgene) adults diet supplemented with tetracycline. This was likely because *tTA* was expressed at an order of magnitude lower level in ovaries from DR5#4 than DR3#4 females. These results highlight the importance of obtaining gene promoters that have high activity early in development but low expression in adult ovary in order to build TSS that do not require addition of tetracycline to the adult diet.

In *L. cuprina*, the *Lcact5C* gene promoter was active in most cells throughout development but showed highest activity in gut in third instar (Fig. 2). Similarly, the distal promoter of the *Dmact5C* gene is most active in the proventriculus and midgut^30^ and in transgenic mosquitoes, a GFP reporter gene driven by the *Dmact5C* promoter retains the tissue specificity as expression was mostly detected in gut tissue^31–33^. However, the overall level of expression of the red fluorescent protein marker driven by the *Lcact5C* promoter in *L. cuprina* was less than what we typically observe in lines that express fluorescent protein marker genes under the control of the *Lchsp83* gene promoter^3, 5^. Since the *Lcact5C* gene appears to be transcribed at high levels in *L. cuprina*, as in *D. melanogaster* ^18^, it would appear that the *Lcact5C* promoter is missing some positive regulatory elements. For the *Dmact5C* distal promoter, positive regulatory elements were found close to the transcription start site and over 2 kb upstream^34^. Further, the mostly heterochromatic environment of the *L. cuprina* X chromosome^35^ could have contributed to low tTA expression in the two viable DR5 lines, which were both X-linked. However, for assembling a TSS the relatively low activity of the *Lcact5C* promoter was likely advantageous as high levels of tTA protein are deleterious^5^ and indeed we were unable to make TSS using the DR4 driver^36^ (Yan and Scott, unpublished), which has the strong *C. hominivorax hsp83* gene promoter controlling tTA expression.

In assembling a two-component TSS it would be ideal if the promoter driving tTA expression was only active at the embryo stage. However, identifying such a promoter is not a trivial task as genes expressed at the embryo stage are often expressed again at later developmental stages^18^. In this study we have shown that it is not necessary to identify an embryo-specific promoter to build an effective TSS. Any detrimental effects from tTA expression at later stages can be minimized through addition of tetracycline to the diet. If genomic resources of a target species are limited, identifying a common constitutive promoter would be a relatively easier task than finding an ideal early promoter. The gene promoter could be from the target species itself or a related species. For the latter, the promoter may have lower activity in the target species but this could be desirable if it is a strong promoter as high levels of tTA expression are deleterious. Simplifying TSS development would be advantageous if resources are limited or if a strain needs to be developed quickly to respond to an outbreak of a pest species (e.g., an invasive species) in an area. TSS developed using this strategy will likely require optimization of a tetracycline-feeding scheme for the adults (as for DH3 and DH4 in this study), but the approach holds good promise to build effective sexing strains that would cost less to rear than a bisexual strain (release generation).

## Methods

### Fly rearing and germ-line transformation

The LA07 wild type strain of *L. cuprina* was maintained and *piggyBac*-mediated germ-line transformation performed as previously described^5^. In brief, adults were kept in mesh cages at 22 °C and fed a sugar/water/protein biscuit diet. Larvae were raised on 93% ground beef at 27 °C and pupae were kept in a 27 °C incubator until eclosion. The transgenic strains were bred to homozygosity by selecting for brightly fluorescing larvae. The amount of any residual tetracycline in the ground beef is unknown but the maximum residue limits for tetracycline, oxytetracycline or chlortetracycline in cattle muscle is 200 μg/kg^37^, which is well below the level (100 μg/mL) that was used to maintain double homozygous strains. The nucleotide sequences flanking a transgene were determined using inverse PCR as previously described^5^. For qRT-PCR analysis, adult DR3#4 or DR5#4 females were anesthetized and dissected in Ringer solution. For each sample, three ovary pairs or three guts were combined in a 1.5 mL tube, then immediately snap frozen in liquid nitrogen. Several samples from each line were stored at −80 °C.

### Female lethality test and embryo-specific lethality assessments

To assess female lethality in a double heterozygous condition, 8 newly emerged males from one homozygous DR3 and 8 newly emerged virgin females from one homozygous effector line, or 8 newly emerged virgin females from DR5#4 (X-linked) and 8 newly emerged males from one homozygous effector line, were put in one bottle and kept on tetracycline-free adult diet for 8 days. Then embryos of 24 h egg lay intervals were reared on tetracycline-free raw ground beef (93% protein and 7% fat) and the number of adult males and females were counted.

To make double homozygous (DH) strains, homozygous virgin females from the EF1 and EF3 lines^6^ were crossed with homozygous males from DR3 and DR4 lines. Since the DR5#4 line is X linked, homozygous DR5#4 virgin females were crossed with homozygous males from EF1 and EF3 lines. The double heterozygous offspring were allowed to interbreed and their progeny screened to select only individuals homozygous for both the driver and effector construct (double homozygous) by epifluorescence microscopy based on fluorescence intensity. Adult flies in all crossings were maintained on 100 μg/mL tetracycline water to repress female lethality. For testing lethality, DH3 and DH4 adults were fed with water containing 3 μg/mL tetracycline for 2 days then switched to tetracycline-free water for 6 days and the offspring raised on diet that lacked tetracycline. DH5 adults were fed water without tetracycline from the day of emergence. Controls were maintained on diet supplemented with 100 μg/mL tetracycline throughout development.

To assess the embryo-specific lethality, 1000 embryos from the 1^st^ egg lay of wild type and DH strains were collected and reared on tetracycline-free (−M) or 100 μg/mL tetracycline (+M) raw ground beef. Then the number of 1^st^ instar, 3^rd^ instar, pupae, adult males and females were counted. All lethality tests were done in triplicate.

### Promoter isolation and plasmid construction

High molecular weight genomic DNA from *L. cuprina* was prepared as previously described^36^. ‘Genome walker’ libraries were prepared from the genomic DNAs using the Universal GenomeWalker kit (Takara) according to the manufacturers instructions. PCR was then performed using the gene-specific primers listed in Table S1. To assemble the construct to test the *Lcactin5C* promoter for activity, RFPto, a version of the *tdTomato* gene^25^ that was codon-optimized for *L. cuprina*, was excised from pUC57-RFPto (Genscript) and inserted into pBC-SV40 pA using unique BamHI and StuI sites. The *Lcactin5C* promoter fragment was amplified from *L. cuprina* genomic DNA using the actinproF-NotI and actinproR-BamHI primer pair (Table S1), and inserted into pBC-RFPto-SV40 using unique NotI and BamHI sites. Then the *Lcactin5C* promoter- RFPto-SV40 cassette were excised by NotI and PspOMI and ligated with the pB[Lchsp83-ZsGreen] vector cut by NotI.

To assemble the driver constructs, gene promoter fragments were amplified from genomic DNA using the primers listed in Table S1 and cloned into pGEM-T (Promega, Madison, WI, USA). After confirmation of the nucleotide sequences, the *Lsspt* promoter was amplified and ligated to pBS- tetO_21_-Dmhsp70 core promoter-tTAV-SV40 (pBS-FL1)^5^ using unique NotI and NcoI sites. This essentially replaced the *tetO*_*21*_-*Dmhsp70* enhancer-promoter with the *Lsspt* promoter. The *Lcactin5C* promoter was amplified by PCR, digested with NotI and NcoI and then ligated to pBS-Lsbnk-tTAopt-p10 ref. 6 that had been cut with the same enzymes. The promoter-tTA-SV40 cassettes were excised using unique XhoI and NotI sites and cloned into the unique XhoI and PspOMI sites in the *piggy*Bac transformation vectors pB[*Lchsp83*-ZsGreen]^3^.

### RNA isolation and RT-PCR analysis

Total RNA was extracted from all life stages using the RNeasy® Mini Kit (QIAGEN) according to the manufacturer’s instructions. Isolated RNA was subsequently treated with RNase-free DNase (QIAGEN). Five μg RNA was used to synthesize cDNA using Superscript III First Strand Synthesis Supermix (Invitrogen) following the manufacturer’s instructions. A control without reverse transcriptase (−RT) was included for each treatment. PCR reactions were assembled using Advantage 2 Polymerase Mix (Clontech) and subjected to the following thermal cycling parameters: initial denaturation for 3 min at 94 °C, 34–38 cycles of (94 °C for 30 s, 52–63 °C for 30 s, 72 °C for 1 min), final extension for 5 min at 72 °C. The annealing temperature for *tTAv*, *tTAo*, *Lcspt*, *Lcact5C* and *LcGST1* primer pairs were 60 °C, 60 °C, 54 °C, 63 °C and 52 °C, respectively. To determine sex, RT-PCR was performed with a *Lctra* primer pair as previously described^6^. qRT-PCR and data analysis was performed as previously described, with *Lc-alphatubulin* (*Lc-atub*) serving as the reference gene^35, 38^.

### Statistical analysis

The Chi-square test and Fisher’s exact test were used to statistically analyze the +/− tetracycline viability data (number flies, not ratios) using the GraphPad Prism 5 and JMP programs.

## Electronic supplementary material

Supplementary Figure Legends

Supplementary Figures

Table S1
