# Supplementary material for: Building early-larval sexing systems for genetic control of the Australian sheep blow fly Lucilia cuprina using two constitutive promoters
Source: Sci Rep. 2017 May 31;7:2538. doi: 10.1038/s41598-017-02763-4 (PMC5451413; doi:10.1038/s41598-017-02763-4)
Supplement: Supplementary file 1 — Supplementary Figure Legends [file 41598_2017_2763_MOESM1_ESM.doc]

**Supplementary Figure Legends**

**Fig. S1**. ***L. sericata* SPITTING IMAGE (SPT) protein alignment.** *L. sericata* SPT (LsSPT) was aligned with SPT protein sequences from *Tribolium castaneum* (TcasSPT), *Aedes aegypti* (AaegSPT), *Anopheles gambiae* (AgamSPT), *Culex quinquefasciatus* (CquiSPT), *Musca domestica* (MdSPT), *D. grimshawi* (DgriSPT), *D. virilis* (DvirSPT), *D. pseudoobscura pseudoobscura* [DpseSPT] and *D. melanogaster* [DmelSPT]. Also included in the alignment are SRY-alpha protein sequences from *D. virilis* (DvSRYalpha), *D. pseudoobscura pseudoobscura* (DpseSRYalpha), *D. suzukii* (DsuzSRYalpha) and *D. melanogaster* (DmSRYalpha). Identical amino acids are dark-shaded and similar amino acids are light-shaded.

**Fig. S2. Phylogenetic analysis of dipteran SPT and SRY-alpha proteins.** An unrooted neighbor-joining tree was constructed with SPT and SRY-alpha protein amino acid sequences. Bootstrap values (1000 replicates) are shown on the nodes of the tree. Species abbreviations are the same as in Fig S1.

**Fig S3.** ***L. cuprina act5C* promoter nucleotide sequence.** The first exon is underlined and a box highlights the TATA regulatory element 30 bp upstream of the likely start of transcription.

**Fig S4. tTAv expression in 2-3h embryos from selected DR3 lines.** qRT-PCR analysis was performed on RNA isolated from 2-3h embryos from DR3 lines. *tTAv* expression was normalized to the zygotic cellularization gene *Lcbnk* (blue) that is strongly expressed in embryo at 2-3h of development (Edman et al, 2015). The experiment was not replicated.

**Fig. S5. *tTAo* expression in the DR5#2 line at different developmental stages.** RNA was isolated from larvae, pupae and adult females from the DR5#2 line and analyzed by RT-PCR. Amplification product sizes as listed for Figure 3.
