# Supplementary material for: Building early-larval sexing systems for genetic control of the Australian sheep blow fly Lucilia cuprina using two constitutive promoters
Source: Sci Rep. 2017 May 31;7:2538. doi: 10.1038/s41598-017-02763-4 (PMC5451413; doi:10.1038/s41598-017-02763-4)
Supplement: Supplementary file 2 — Supplementary Figures [file 41598_2017_2763_MOESM2_ESM.pdf]

Formatted Alignments

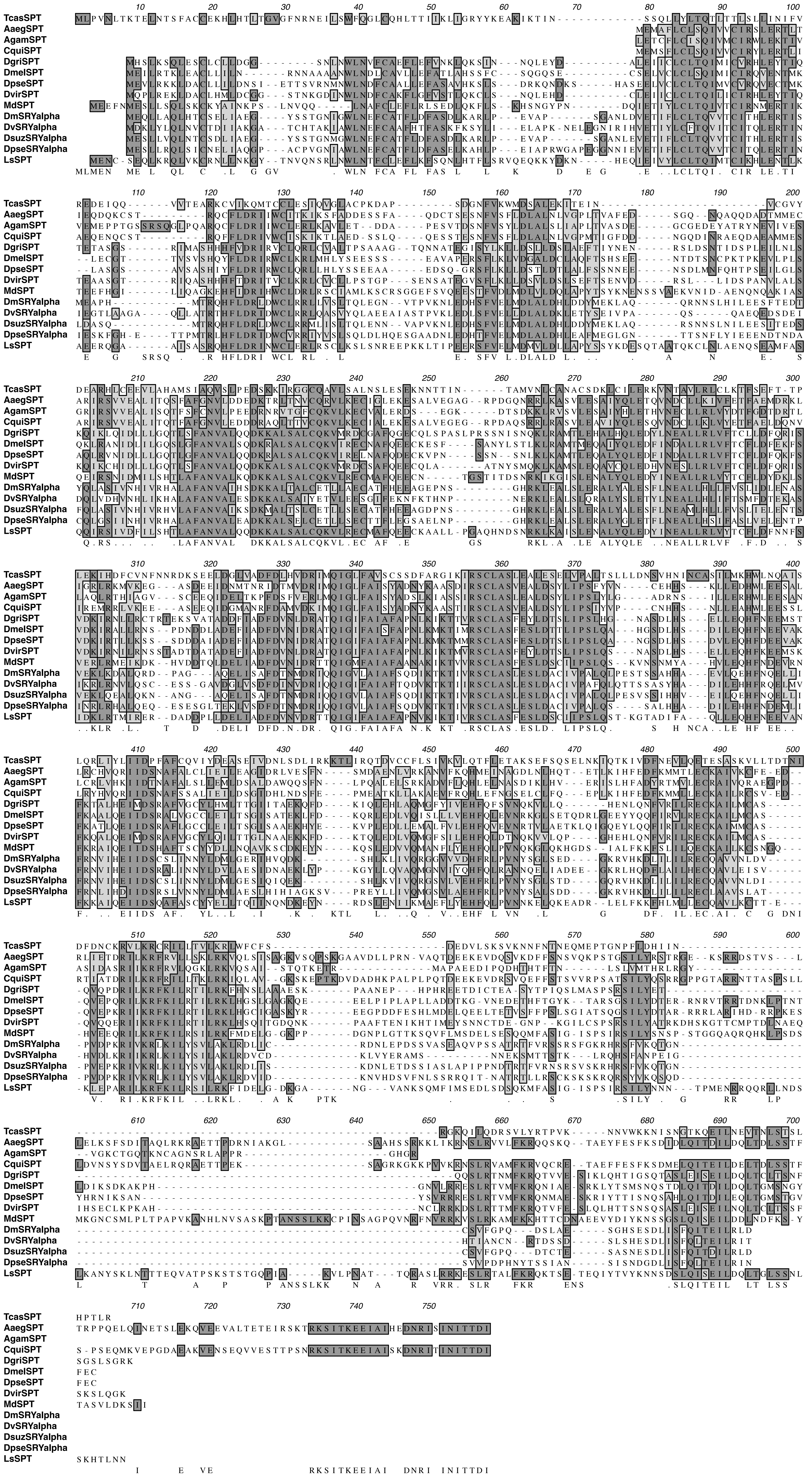

Method: Neighbor Joining; Bootstrap (1000 reps); tie breaking = Systematic
Distance: Uncorrected ("p")
Gaps distributed proportionally

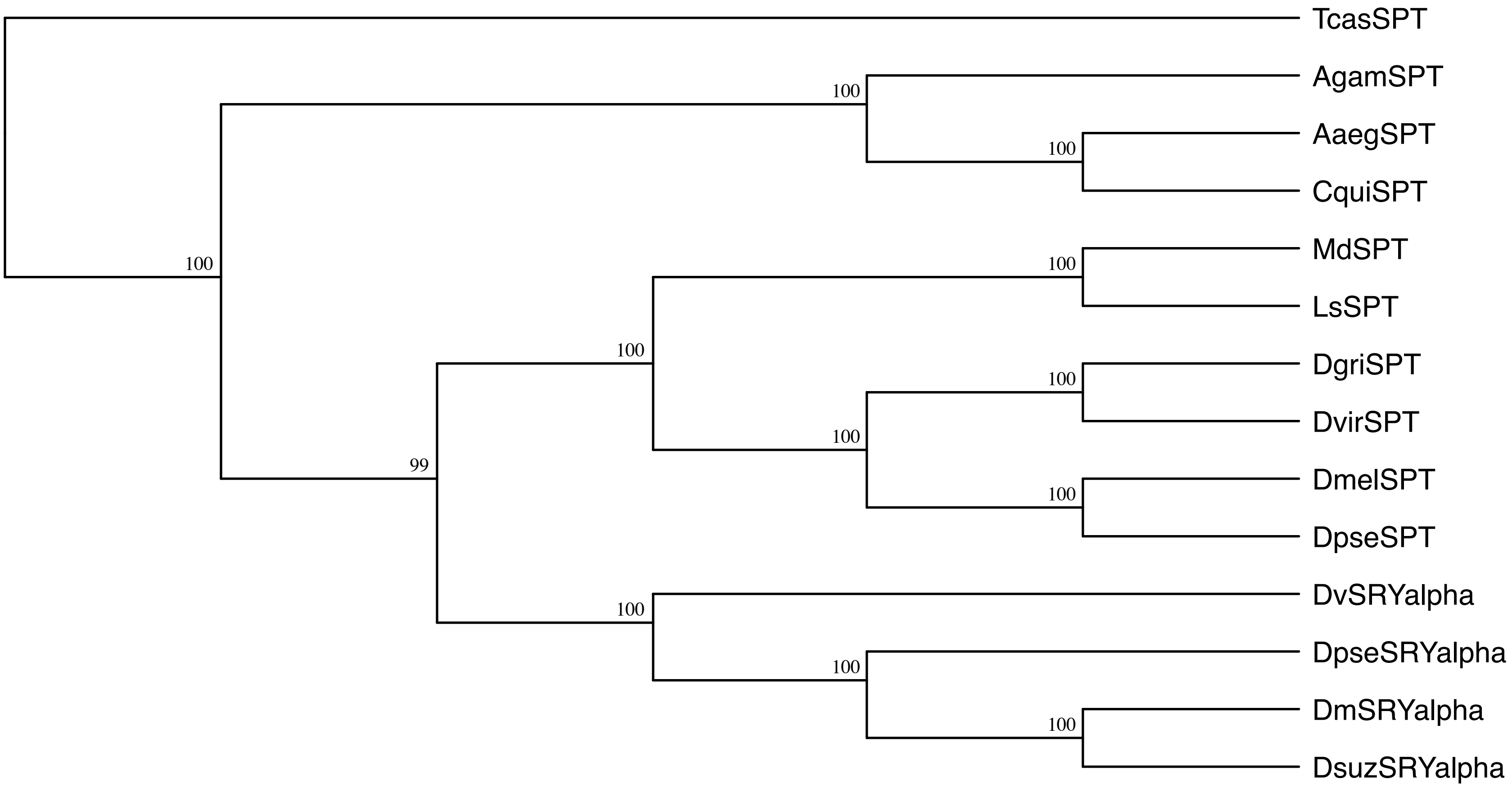

```
         10        20        30        40        50        60        70        80        90       100       110
AAATAAATAAAAATGAAAAAATTCAGACGGCGATTAAACTTATCGGTTTTCGTTGCGCGAGTGTGTGCGTGAATCGTGTGTTGTATTTGTTGTATTTTCAATGCGGGTTTA

       120       130       140       150       160       170       180       190       200       210       220
TTACTCATTTCTCGTTTCTCATTTCTCATTACTCAAAATACGAAATTCTCTAGAAATATGGCAACAATGCTGCACGTTTTGTGCATCCAATGGATGCCATCCATACCCATA

      230       240       250       260       270       280       290       300       310       320       330
TATGGTAAAATGTCTGAAAAACACTAGAGAACCCATATGAAACGCGTTTTGATAAGGGTAGCCGCCTTTATAAGCGCTTGTAACAACGAGTTTACTATCATTAAAGTTTG

     340       350       360       370       380       390       400       410       420       430       440
CGTTCTTGTGCTGTGTGGATACCAACGCGAGACCTCCCAAACCAACAAAAAGACTTAATTTATTTATTTAATTTTAGTAATTTAATAAAACACAAAGTAAGTATTTTGTAA

    450       460       470       480
TAAGCAACAGCAGCGACAAACAATAAAACTAGTGAAAAAT
```

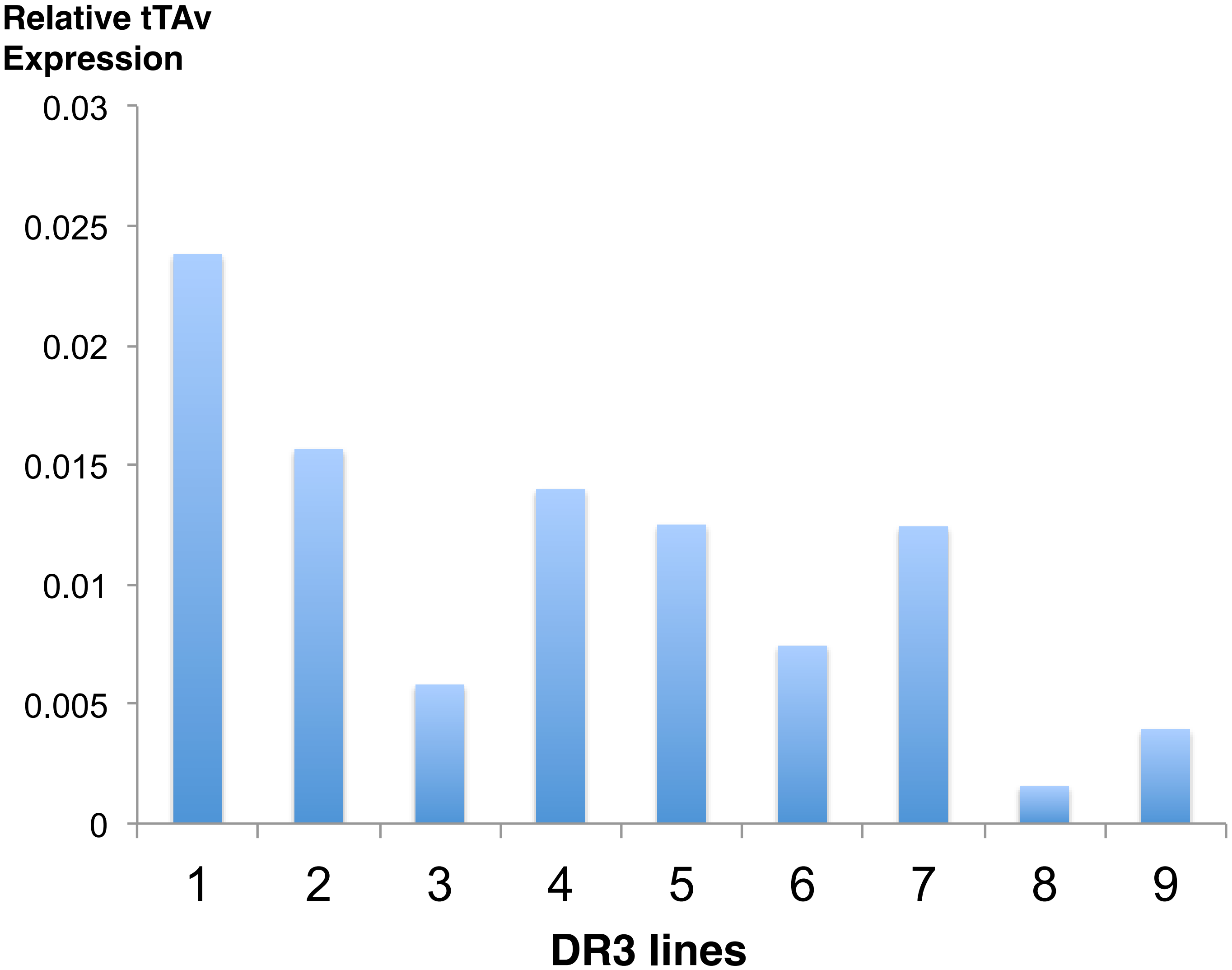
Relative tTAv
Expression
0.03
0.025
0.02
0.015
0.01
0.005
0
1
2
3
4
5
6
7
8
9
DR3 lines

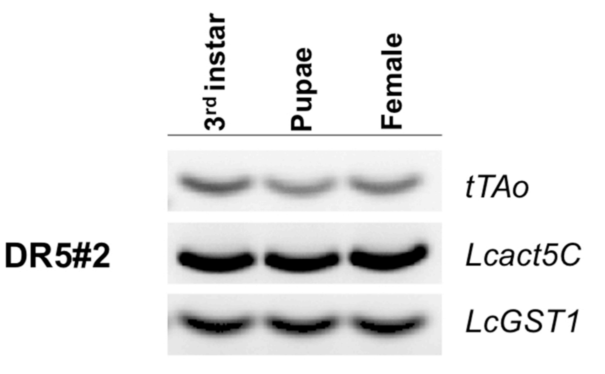
DR5#2
3rd instar
Pupae
Female
tTAo
Lcact5C
LcGST1
