# Supplementary material for: Building early-larval sexing systems for genetic control of the Australian sheep blow fly Lucilia cuprina using two constitutive promoters
Source: Sci Rep. 2017 May 31;7:2538. doi: 10.1038/s41598-017-02763-4 (PMC5451413; doi:10.1038/s41598-017-02763-4)
Supplement: Supplementary file 3 — Table S1 [file 41598_2017_2763_MOESM3_ESM.doc]

Table S1 Primer sets used

| Primer name | Primer sequence（5’-3’） |
| --- | --- |
| pBacRNA-F | CCTAATACGACTCACTATAGGGAGACTTATTATATATATATTTTCTTGTT |
| pBacRNA-R | ATCGGTCTGTATATCGAGGTTTATTTATTAATTTGAA TAGATATT |
| Lsspt-GSP1 | AAATTCGGCGCAATTTTATAGATGCTGTTGCTGTTTTTATTTG |
| Lsspt-GSP2 | CGGTTTTCAAGGCGAATTTAATAAGGTTACAACAGCATTGAT |
| Lsspt-GSP3 | TACTATTGTGAAGACTATACTTGATTCAGCGTATCA |
| Lsspt-GSP4 | GTTGTATTAAAAATCATGAATAAAAAAAATTTCAAAATACCGAA |
| Lsspt-GSP5 | AAAAGTAGTGTGTAATTCAACAGGAAAACGTCTC |
| Lsspt-GSP6 | TATTTTCTTTAGCAGCAGTCGTAGCAA |
| LssptpromF | ATAGCGGCCGCCTGGTAAACGAAAGATCGTAAAATGATAA |
| LssptpromR | CTGCCATGGTTGTATATGATTTTTGTTTTATTT |
| LcAc5C-GSP1 | TAAGTCTTTTTGTTGGTTTGGGAGGTC |
| LcAc5C-GSP2 | CAGCACAAGAACGCAAACTTTAATGAT |
| LcAc5C-GSP3 | GCAGCAACTTCTTCGTCGCACATTT |
| LcAc5C-GSP4 | CTAAGGCAGCAACTTCTTCGTCGCA |
| actinproF-NotI | GCGGCCGCTTCTATTTTTCAAACTAGGATTAATTTTCATG |
| actinproR-BamHI | GGATCCACTAGTAAGGCAGCAACTTCTTCGTCGC |
| actinproR-NcoI | TCGCCCATGGTGTTTGCTGCAAAATAAATAATTAAAAAAAGTTAATATAATATTGCATGGA |
| tTAo-F-AvrII | AAGCCTAGGCCATGGTGAGCAGATTGGATAAAAGCAAAGTG |
| tTAo-R-NgoMIV | ATAGCCGGCAAAGATCCAAGCTTATCATCCACCATATTC |
| Lcspt-RT-F | AACAATGATAAGGGAACGTGATGC |
| Lcspt-RT-R | GCCTGACATTCTAGCAGCATTAAG |
| Lcactin-RT-F | GCTGCCTTAGTTGTTGATAACGGTTCCGGTATGT |
| Lcactin-RT-R | GGGCATAACCTTCATAGATTGGTACGGTGTGGGA |
| tTAV-RT-F | TCTTGCGTAATAATGCCAAATCCTTCCG |
| tTAV-RT-R | CCAACACACAGCCCAATGTAAAATGACC |
| tTAo-RT-F | TGTTGAATGAAGTGGGTATTGAAGGATTGACTACTCG |
| tTAo-RT-R | CCAAAGGGCAAAAGTGGGTGTGATGTCTATC |
| LcGST1-F | GCCAGTGTCAGCACCTTTG |
| LcGST1-R | GCAACCTTCCCAGTTTTCATC |
| LctraF | CACAACAACTGCTTATCATCGGCAACAACAACTAC |
| LctraR-qRT | TTATTAGTTTTACAGCCCGTATTGCG |
| P10pA-F | CCGAAGCTTCTAGAATGAATCGTTTTTAAAATAACAAATCAATTGTT |
| P10pA-R | AATCTCGAGGCGCGCCGGCCGTTAACTCGAATCGCTAT |
| Lc α-tubulin-FW | GTGATTTGGCCAAGGTACAACGTG |
| Lc α-tubulin-RV | CGACGTACCAGTGGACGAAAGC |
| LcVasa FW | TAATAGTATCACCCACACGCGAAT |
| LcVasa RV | TGACTACCACCACGACCAATATTT |
| LcSer6 FW 3 | TCCTGTTGGTGGAATAGTGACAAT |
| LcSer6 RV 3 | TTACAATCGTTTCGGGAAATTGCA |
